# Infant Mortality Attributable to Birth Defects — United States, 2003–2017

**DOI:** 10.15585/mmwr.mm6902a1

**Published:** 2020-01-17

**Authors:** Lynn M. Almli, Danielle M. Ely, Elizabeth C. Ailes, Rahi Abouk, Scott D. Grosse, Jennifer L. Isenburg, Debra B. Waldron, Jennita Reefhuis

**Affiliations:** ^1^National Center on Birth Defects and Developmental Disabilities, CDC; ^2^National Center for Health Statistics, CDC; ^3^Cotsakos College of Business, William Paterson University, Wayne, New Jersey; ^4^American Academy of Pediatrics, Itasca, Illinois.

Birth defects are a leading cause of infant mortality in the United States, accounting for 20.6% of infant deaths in 2017 (*1*). Rates of infant mortality attributable to birth defects (IMBD) have generally declined since the 1970s (*1*–*3*). U.S. linked birth/infant death data from 2003–2017 were used to assess trends in IMBD. Overall, rates declined 10% during 2003–2017, but decreases varied by maternal and infant characteristics. During 2003–2017, IMBD rates decreased 4% for infants of Hispanic mothers, 11% for infants of non-Hispanic black (black) mothers, and 12% for infants of non-Hispanic white (white) mothers. In 2017, these rates were highest among infants of black mothers (13.3 per 10,000 live births) and were lowest among infants of white mothers (9.9). During 2003–2017, IMBD rates for infants who were born extremely preterm (20–27 completed gestational weeks), full term (39–40 weeks), and late term/postterm (41–44 weeks) declined 20%–29%; rates for moderate (32–33 weeks) and late preterm (34–36 weeks) infants increased 17%. Continued tracking of IMBD rates can help identify areas where efforts to reduce IMBD are needed, such as among infants born to black and Hispanic mothers and those born moderate and late preterm (32–36 weeks).

Linked birth/infant death records for infants aged <1 year born to U.S. residents (excluding U.S. territories) from 2003, the first year of the birth certificate revision,* through 2017 were obtained from the National Vital Statistics System.^†^ Most (98.4%–99.6%) infant death records were linked to their corresponding birth certificates (percentage of matched records varied by year). To account for nonlinkage, the linked birth/infant death file was weighted by the proportion of death certificates unlinked to their corresponding birth certificate each year by state and age at death. Last menstrual period date obtained from the birth certificate was used to calculate gestational age at birth. Records of 7.8% of infant deaths and 1.4% of live births with missing or implausible gestational age (i.e., gestational age <20 weeks, >44 weeks, or incompatible with birthweight) (*4*) were excluded. Maternal race/ethnicity was obtained from the birth certificate where multiple-race/Hispanic-origin responses were converted to single bridged-race categories (*5*). *International Classification of Diseases, Tenth Revision* was used to identify deaths with a major birth defect listed as the underlying cause of death (codes Q00.0–Q99.9). The following conditions were not considered causes of IMBD: undescended testicles (Q53.1, Q53.2, and Q53.9); cardiovascular conditions not considered to be structural heart defects (Q27.0–Q28.9); and preterm births (20–36 weeks) with an underlying cause of death considered to be a complication of prematurity (lung hypoplasia [Q33.6]). In addition, underlying causes of death listed as persistent foramen ovale (Q21.1) and patent ductus arteriosus (Q25.0) were excluded for all preterm births and for infants born at term/postterm (37–44 weeks) with an age of death <28 days (neonatal).

IMBD rates and 95% confidence intervals (CIs) were calculated for each year and stratified by maternal race/ethnicity (white, black, or Hispanic), maternal age at delivery (<20 years, 20–34 years, or >34 years), infant sex, gestational age (20–27, 28–31, 32–33, 34–36, 37–38, 39–40, or 41–44 weeks), and infant age at death (neonatal [<28 days] or postneonatal [28–364 days]). Births from other racial/ethnic groups were excluded from race/ethnicity analyses but were included in the total counts. Overall and for each stratum, percent changes in IMBD rates were calculated by dividing the difference between the rates in 2003 and 2017 by the rate in 2003, and then multiplying by 100. References to decreasing or increasing trends during 2003–2017 are statistically significant and were assessed using the Cochran–Armitage test for trend. SAS statistical software (version 9.4; SAS Institute) was used for analyses.

The 2003–2017 linked birth/infant death data included 60,036,305 live births and a weighted total of 384,223 infant deaths (349,049 after exclusions). A birth defect was listed as the underlying cause of death for 70,954 (20.3%) infant deaths during 2003–2017, ranging from 19.5% (4,898 of 25,069) in 2003 to 20.7% (4,186 of 20,179) in 2017. IMBD rates decreased 10% from 2003 (12.2 per 10,000 live births [95% CI = 11.9–12.6]) to 2017 (11.0 per 10,000 live births [95% CI = 10.7–11.3]) (Table) (Figure 1).

**TABLE:** Rates of infant mortality attributable to birth defects (IMBD) in 2003 and 2017 and percentage change, by maternal race/ethnicity, maternal age at delivery, infant sex, gestational age, and infant age at death — United States, 2003–2017

Characteristic	IMBD rates per 10,000 live births (95% CI)		% Change* 2003–2017
	2003	2017	
	Total IMBD cases = 4,897; total infant births = 3,998,383	Total IMBD cases = 4,186; total infant births = 3,809,747	
**Total IMBD**	**12.2 (11.9–12.6)**	**11.0 (10.7–11.3)**	**−10^§^**
**Maternal race/ethnicity^†^**			
White, non-Hispanic	11.3 (10.9–11.8)	9.9 (9.4–10.3)	−12^§^
Black, non-Hispanic	14.9 (13.8–15.9)	13.3 (12.4–14.3)	−11^§^
Hispanic	13.0 (12.2–13.7)	12.5 (11.8–13.2)	**−**4^§^
**Maternal age at delivery (yrs)**			
<20	13.3 (12.2–14.4)	12.9 (11.3–14.5)	**−**3
20–34	11.5 (11.1–11.9)	10.1 (9.7–10.4)	**−**12^§^
>34	15.5 (14.5–16.5)	14.5 (13.5–15.4)	**−**6^§^
**Infant sex**			
Male	12.8 (12.3–13.3)	11.0 (10.5–11.5)	**−**14^§^
Female	11.7 (11.2–12.2)	11.0 (10.5–11.4)	**−**6^§^
**Gestational age (wks)**			
20–27	198.5 (181.5–215.6)	158.8 (142.7–175.0)	**−**20^§^
28–31	110.0 (99.9–120.0)	104.0 (93.8–114.1)	**−**5
32–33	58.2 (52.1–64.3)	67.9 (61.0–74.8)	17^§^
34–36	25.4 (23.8–27.1)	29.6 (27.7–31.5)	17^§^
37–38	10.5 (9.9–11.1)	10.5 (9.9–11.2)	0
39–40	5.9 (5.5–6.2)	4.2 (3.9–4.5)	**−**29^§^
41–44	7.1 (6.4–7.7)	5.3 (4.6–5.9)	**−**25^§^
**Infant age category at death^¶^**			
Neonatal	8.5 (8.2–8.7)	7.9 (7.6–8.2)	**−**7^§^
Postneonatal	3.8 (3.6–4.0)	3.1 (2.9–3.3)	**−**18^§^

**Abbreviation:** CI = confidence interval.

* Overall percent change was calculated as 100 × ((IMBDrate_2003_ - IMBDrate_2017_)/IMBDrate_2003_).

^†^ Excludes infants born to mothers of other reported racial/ethnic groups and other/unknown maternal race/ethnicity.

^§^ Significant trend in IMBD rates during 2003–2017 using the Cochran-Armitage test for trend.

^¶^ Neonatal: <28 days; postneonatal: 28–364 days.

**FIGURE 1:**
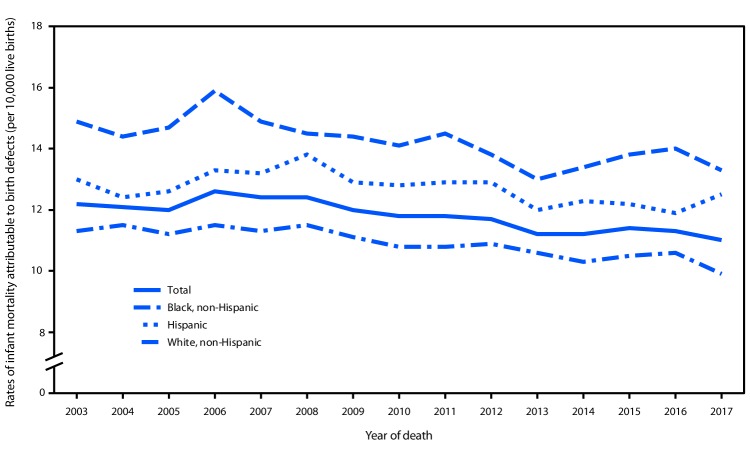
Rates of infant mortality attributable to birth defects, by maternal race/ethnicity — United States, 2003–2017

Significant trends in IMBD during 2003–2017 were observed across most maternal racial/ethnic, maternal age, infant sex, gestational age, and infant age at death categories (Table). Rates decreased 4% during 2003–2017 for infants of Hispanic mothers (from 13.0 infant deaths per 10,000 live births to 12.5), 11% for infants of black mothers (from 14.9 to 13.3), and 12% for infants of white mothers (from 11.3 to 9.9) (Table) (Figure 1). During 2003–2017, rates were consistently higher among infants of black mothers and lowest among infants of white mothers (Figure 1). Trends varied by maternal age: rates for infants of mothers aged <20 years were stable from 2003 (13.3) to 2017 (12.9), but rates decreased 12% for infants of mothers aged 20–34 years (from 11.5 to 10.1) and 6% for mothers aged >34 years (from 15.5 to 14.5). IMBD rates decreased 14% during 2003–2017 for male infants (from 12.8 to 11.0) and 6% for female infants (from 11.7 to 11.0). Among extremely preterm infants (20–27 weeks), rates declined 20% (from 198.5 to 158.8); however, significant 17% increases occurred among infants born at 32–33 weeks (from 58.2 to 67.9) and 34–36 weeks (from 25.4 to 29.6) (Figure 2) (Table). IMBD rates declined 29% among infants born at 39–40 weeks (from 5.9 to 4.2) and 25% among infants born at 41–44 weeks (from 7.1 to 5.3). Since 2003, rates were stable among infants born at 28–31 weeks (from 110.0 to 104.0) and 37–38 weeks (10.5 in both years). Trends in IMBD also differed by infant age category at death (neonatal or postneonatal); rates in both categories declined significantly: a 7% decline (from 8.5 to 7.9) in neonatal rates and an 18% decline (from 3.8 to 3.1) in postneonatal rates.

**FIGURE 2:**
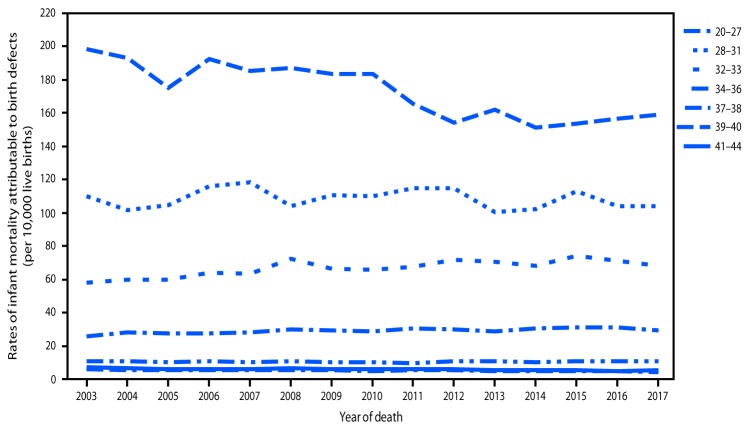
Rates of infant mortality attributable to birth defects, by infant gestational age at birth (weeks) — United States, 2003–2017

## Discussion

Rates of IMBD decreased 10% during 2003–2017 overall and across the categories of maternal race/ethnicity, infant sex, and infant age at death. Although rates declined among infants of Hispanic, black, and white mothers, racial/ethnic disparities persisted. The IMBD rate was 32% (2003) and 34% (2017) higher among infants born to black mothers than that among those born to white mothers and 15% (2003) and 26% (2017) higher among infants born to Hispanic mothers than among those born to white mothers. Across gestational age categories, declines in IMBD rates were limited to infants born at 20–27 and 39–44 weeks, and rates increased for those born at 32–36 weeks and were stable for those born at 28–31 and 37–38 weeks.

The decline in IMBD could be influenced by improvements in prenatal care, birth defects prevention measures, and improvements in medical care of infants with birth defects, in addition to factors influencing the overall infant mortality rate. The observed differences in IMBD rates by race/ethnicity might be influenced by access to and utilization of health care before and during pregnancy, prenatal screening, losses of pregnancies with fetal anomalies, and insurance type (*6*,*7*). Although IMBD rates for extremely preterm and late term/postterm infants significantly decreased over the 15-year period, rates among moderate and late preterm infants increased. These trends could be influenced by the quantity and quality of care for infants born before 30 weeks gestation, compared with that of those born closer to term (*8*).

The findings in this report are subject to at least four limitations. First, deaths for which birth defects were listed as a contributing but not the underlying cause of death (13%–15% during 2003–2017)^§^ were not included, possibly resulting in an underestimation of IMBD. Second, cause of death classifications might vary by the maternal and infant factors considered in this report. Third, gestational age categories in this report were calculated from date of the last menstrual period and thus are subject to misclassification. Gestational age categories determined by obstetric estimates have shown increased validity and are the preferred measure (*9*), but these were not available for the full period under study. Finally, examining trends in IMBD rates by specific type of birth defect was beyond the scope of the study, but could provide additional information to inform prevention efforts.

Birth defects occur in approximately 3% of births (*10*) yet are a leading cause of infant mortality (*1*). The results from this analysis can inform future research into areas where efforts to reduce IMBD rates are needed, such as among infants born to black and Hispanic mothers and those born moderate/late preterm (32–36 weeks).

**Summary**

**What is already known about this topic?**

Rates of infant mortality attributable to birth defects have been declining since 1970.

**What is added by this report?**

During 2003–2017, rates of infant mortality attributable to birth defects declined 10% overall, including among infants of Hispanic mothers (4%), non-Hispanic black mothers (11%), and non-Hispanic white mothers (12%); however, racial/ethnic disparities remained. Rates decreased for extremely preterm infants (20–27 completed gestational weeks) and late term/postterm infants (39–44 weeks) but increased for moderate/late preterm infants (32–36 weeks).

**What are the implications for public health practice?**

Continued tracking of rates of infant mortality attributable to birth defects by maternal and infant characteristics can help identify areas where efforts to reduce mortality rates are needed.

## Footnotes

* https://www.cdc.gov/nchs/nvss/revisions-of-the-us-standard-certificates-and-reports.htm.

† https://www.cdc.gov/nchs/data_access/vitalstatsonline.htm.

§ Data are from CDC WONDER’s Multiple Cause of Death Files, 1999–2017, as compiled from data provided by the 57 vital statistics jurisdictions through the Vital Statistics Cooperative Program. https://wonder.cdc.gov/mcd-icd10.html.
